# Increased PADI4 expression in blood and tissues of patients with malignant tumors

**DOI:** 10.1186/1471-2407-9-40

**Published:** 2009-01-30

**Authors:** Xiaotian Chang, Jinxiang Han, Li Pang, Yan Zhao, Yi Yang, Zhonglin Shen

**Affiliations:** 1 Key Laboratory for Bio-drugs of Ministry of Health, Research Center For Medicinal Biotechnology, Shandong Academy of Medical Sciences, Jingshi Road 89, Jinan, Shandong, 250062, PR China; 2 Shandong University 2nd Hospital, Shandong University Medical School, Jinan, Shandong, PR China; 3 Yankuang Hospital of Yanzhou, Yanzhou, Shandong, PR China

## Abstract

**Background:**

Peptidylarginine deiminase type 4 (PAD4/PADI4) post-translationally converts peptidylarginine to citrulline. Recent studies suggest that PADI4 represses expression of p53-regulated genes via citrullination of histones at gene promoters.

**Methods:**

Expression of PADI4 was investigated in various tumors and non-tumor tissues (n = 1673) as well as in A549, SKOV3 and U937 tumor cell lines by immunohistochemistry, real-time PCR, and western blot. Levels of PADI4 and citrullinated antithrombin (cAT) were investigated in the blood of patients with various tumors by ELISA (n = 1121).

**Results:**

Immunohistochemistry detected significant PADI4 expression in various malignancies including breast carcinomas, lung adenocarcinomas, hepatocellular carcinomas, esophageal squamous cancer cells, colorectal adenocarcinomas, renal cancer cells, ovarian adenocarcinomas, endometrial carcinomas, uterine adenocarcinomas, bladder carcinomas, chondromas, as well as other metastatic carcinomas. However, PADI4 expression was not observed in benign leiomyomas of stomach, uterine myomas, endometrial hyperplasias, cervical polyps, teratomas, hydatidiform moles, trophoblastic cell hyperplasias, hyroid adenomas, hemangiomas, lymph hyperplasias, schwannomas, neurofibromas, lipomas, and cavernous hemangiomas of the liver. Additionally, PADI4 expression was not detected in non-tumor tissues including cholecystitis, cervicitis and synovitis of osteoarthritis, except in certain acutely inflamed tissues such as in gastritis and appendicitis. Quantitative PCR and western blot analysis showed higher PADI4 expression in gastric adenocarcinomas, lung adenocarcinomas, hepatocellular carcinomas, esophageal squamous cell cancers and breast cancers (n = 5 for each disease) than in the surrounding healthy tissues. Furthermore, western blot analysis detected PADI4 expression in cultured tumor cell lines. ELISA detected increased PADI4 and cAT levels in the blood of patients with various malignant tumors compared to those in patients with chronic inflammation and benign tumors. This was consistent with immunohistochemical results. Additionally, PADI4 and cAT levels were significantly associated with higher levels of known tumor markers.

**Conclusion:**

Our results suggest that PADI4 expression is increased in the blood and tissues of many malignant tumors, a finding useful for further understanding of tumorigenesis.

## Background

In a process known as citrullination, peptidylarginine deiminase type 4 (PAD4/PADI4) post-translationally converts peptidylarginine to citrulline. Previously, Cuthbert et al. had found that citrullination by PADI4 prevents arginine methylation of histones H3 and H4 during transcriptional activation of estrogen-responsive genes [1]. Further evidence has confirmed that PADI4 is significantly associated with rheumatoid arthritis (RA), thus playing an important role in the pathogenesis of this disease [2]. We previously detected intense PADI4 expression in tumor cells from various adenocarcinomas, but not in healthy tissues [3]. We also demonstrated co-localization of PADI4 with cytokeratin, an intermediate filament protein that plays a role during cell differentiation and apoptosis [3-6]. To further confirm these findings, we investigated PADI4 expression by immunohistochemistry, real-time PCR and western blot analysis in various benign tumors and non-tumor inflamed tissues in the current study. Due to the relatively low, sometimes undetectable, expression of PADI4 in tumor tissue extracts, we performed western blot following immunoprecipitation in the study. We also examined PADI4 expression in cultured lung adenocarcinoma (A549), ovarian adenocarcinoma (SKOV3), and leukemia (U937) cell lines. Additionally, we investigated PADI4 levels in the blood of patients with various tumors in the present study. Since we previously detected citrullination and subsequent inactivation of antithrombin in the plasma of RA patients [7], we also measured levels of citrullinated antithrombin (cAT) in the blood of patients containing various tumor types. By analyzing levels of PADI4 and cAT, as well as the levels of known tumor markers, we aimed to explore the pathogenic role of PADI4 during carcinogenesis.

## Methods

### Immunohistochemistry

Various malignant tumors, benign tumors and non-tumor inflamed tissues (n = 1673, see Table 1) were collected during excision surgery at several hospitals in the Shandong region of China. Tumor diagnosis was verified by histological methods, and pathological categorization was determined according to the World Health Organization (WHO) classification system. All patients signed informed consents, and this study was approved by the ethics committee of Shandong Academy of Medical Sciences.

**Table 1:** The PADI4 expression in tumors and non-tumor inflamed tissues

tissues	expression	no expression	positivity %
breast cancer	144	6	96
breast fibroadenoma	5	72	6.5
esophageal squamous cell cancer	80	15	84.2
gastric adenocarcinoma	56	10	84.8
rodents	24	38	38.7
gastritis	20	44	31.2
leiomyosarcoma of stomach	7	38	15.6
colorectal adenocarcinoma	24	4	85.7
cholecystitis	0	20	0
appendicitis	20	4	83.3
myoma of uterus	0	60	0
endometrial hyperplasia of uterus	10	66	13.1
endometrial carcinoma	31	1	96.8
cervical polyp	0	20	0
cervicitis	0	20	0
hydatidiform mole	0	20	0
ovarian carcinoma	66	2	97
hydatidiform mole	0	20	0
teratoma	8	40	16.7
primary lymphoma	20	8	71.4
lymphadenitis	0	20	0
skin cancer	20	0	100
intradermal nevus	0	20	0
lipoma	0	20	0
hepatocellular carcinoma	20	0	100
hepatoblastoma	0	20	0
liver cirrhosis	108	52	67.5
hepatic cavernous hemangioma	0	72	0
lung adenocarcinoma	62	0	100
renal cell carcinoma	47	7	87
chondroma	20	0	100
osteosarcoma	50	1	98
bladder carcinomas	20	0	100
metastatic carcinoma	20	0	100
thyroid adenoma	0	40	0
hemangioma	0	20	0
neurofibroma	0	20	0
pleomorphic adenoma of salivary	2	22	8.3
synovitis	0	18	0

Tissue samples were fixed in 10% neutral buffered formalin and embedded in paraffin. Tissue sections were deparaffinized and rehydrated using standard procedures. To increase the intensity of immunostaining, the sections were heated at 95°C for 10 min in citrate buffer (0.01 M, pH 0.6), then incubated overnight at 4°C with anti-PADI4 antibody. Anti-PADI4 was prepared by immunizing rabbits with the synthetic oligopeptide FGDSCYPSNDSRQMH, and immunospecificity was confirmed in a previous study [3]. Immunoreactions were processed using the UltraSensitive TM S-P Kit (Maixin-Bio, China) according to the manufacturer's instructions, and signals were visualized using the DAB substrate, which stains the target protein yellow.

The intensity of immunosignals was evaluated by a previously described protocol from Denkert et al. [8]. For each histological section, the percentage of positive cells was scored as 0 (0%), 1 (10%), 2 (10–50%), 3 (51–80%) and 4 (> 80%), and the staining intensity was scored as 0 (negative), 1 (weak), 2 (moderate) and 3 (strong). The immunoreactive score (IRS) was obtained by multiplying the percentage of positive cells and the staining intensity. Immunohistochemical results with an IRS of 0–1 were considered negative, 1–2 weak and 6–12 positive.

### Immunoprecipitation and western blot analysis

Gastric adenocarcinomas (n = 5), lung adenocarcinomas (n = 5), hepatocellular carcinomas (n = 4), esophageal squamous cell cancers (n = 5), breast cancers (n = 5), and breast fibroadenomas (n = 5) were collected during excision surgery. Normal, healthy tissues located 5 cm away from the corresponding tumors were collected during surgery for control histological examinations. Sample tissues (200 ug) were homogenized with Cell Lysis Solution (Sigma) and centrifuged at 16000 × g for 5 min at 4°C. The supernatant was collected, and protein concentrations were determined using the BCA Protein Assay Kit (Pierce). Immunoprecipitation (IP) was performed using a Protein G Immunoprecipitation Kit (Sigma) according to the manufacturer's instructions. Briefly, an equal amount of lysate from each sample was incubated overnight at 4°C with the PADI4 antibody in the presence of a Protease Inhibitor Cocktail (Sigma). Protein G beads were added to the mixture and incubated for 2 h at 4°C. After thorough washing, the purified PADI4 protein was eluted with 1× Laemmli sample buffer (Sigma). IP samples (10 ul) were separated by SDS-PAGE and then transferred onto PVDF membranes using the Mini-PROTEAN 3 system (Biorad). The membranes were probed with monoclonal anti-PADI4 antibody containing a recombinant fragment of human PADI4 between residues 2–111 (Abcam), and then incubated with sheep anti-mouse IgG conjugated to alkaline phosphatase (Sigma). Immunosignals were visualized with the Protein Detector BCIP/NBT Western Blot Kit (KPL) following the manufacturer's instructions.

We also measured PADI4 expression in total protein extracts from the tumor cell lines A549, SKOV3 and U937. Preparation of protein extracts was performed as described above.

### ELISA

Blood samples were collected from patients with malignant tumors 4–6 days prior to and after tumor excision surgery. Blood from healthy volunteers and from patients with benign tumors or non-tumor inflammation were used as controls. All samples (n = 1121, see Table 2) were collected in Monovette tubes containing 3.8% sodium citrate and centrifuged at 1500 × g for 20 min. Supernatants of anticoagulated plasma were collected and stored at -80°C until use. Plasma samples were diluted 1:20 in 0.05 M carbonate-bicarbonate buffer (pH 9.6) and used to coat 96-well EIA/RIA microplates (Costar) overnight at 4°C. After a brief wash with PBST (8 g NaCl, 0.2 g KCl, 1.15 g NaHPO_4 _and 0.2 g KH_2_PO_4 _per liter, pH 7.4–7.6, 0.1% Tween 20), plates were blocked with 5% non-fat dry milk for 1 h at room temperature. Anti-PADI4 antibody (diluted 1:4000 in PBST) was added to the plates and incubated for 2 h at room temperature. After washing with PBST, plates were incubated with 1:10,000 anti-rabbit IgG conjugated to alkaline phosphatase (Sigma) for 30 min at room temperature. Plates were then washed with PBST, and the signal was developed by adding the Alkaline Phosphatase Yellow (pNpp) Liquid Substrate System for ELISA (Sigma). Absorbance at 405 nm was measured using a spectrophotometer (Synergy HT, Bio-Tek).

### Sandwich ELISA

Anti-human antithrombin monoclonal antibody (Abcam) was diluted 1:5000 in 0.05 M carbonate-bicarbonate buffer (pH 9.6) and used to coat microplates overnight at 4°C. After a brief wash with PBST, the plates were blocked with 5% non-fat dry milk for 1 h at room temperature. Plasma samples diluted 1:20 in PBST were added to the plates and incubated for 2 h at room temperature. After washing with PBST, plates were incubated with 1:4000 rabbit anti-citrulline (Abcam) for 1 h at room temperature. The anti-citrulline antibody was labeled with alkaline phosphatase using the AP Labeling Kit (Roche) according to the manufacturer's instructions. The plates were then washed with PBST, and signals were detected as described above.

### Measure of tumor markers in patient blood samples

CEA levels were measured in all blood samples, AFP levels in liver cancer samples, CA199 levels in digestive tract tumor samples, CA125 levels in ovarian cancer samples, CA153 levels in breast cancer samples, CYFRA21-1 levels in lung cancer samples, and PSA levels in prostate cancer samples. All measurements were performed in a clinical laboratory cooperating with us.

ELISA data were collected from three independent tests. All statistical analyses were conducted using SPSS (version 11.0). The median and range of PADI4 and cAT levels are reported. The Mann-Whitney U-test statistically assessed differences between the groups, and the x^2 ^test was used to examine the association between expression of PADI4, cAT and several tumor markers. P values ≤ 0.05 were considered as statistically significant.

### Real-Time Quantitative PCR

Total RNA was extracted using the TRIzol reagent (Invitrogen) from gastric adenocarcinomas (n = 5), lung adenocarcinomas (n = 5), hepatocellular carcinomas (n = 4), esophageal squamous cell cancers (n = 5), breast cancers (n = 5) and breast fibroadenomas (n = 5), as well as their corresponding healthy tissues. Concentrations of total RNA were determined with a spectrophotometer. Quantitative PCR analyses were performed using the iCycler Real-Time Detection System (Bio-Rad). Gene-specific primers were designed using the PADI4 mRNA sequence in Genbank (NM012387) and were as follows: forward 5'-ctgtggtgttccaagacagc-3' (nt position 871–890) and reverse 5'-gcttggatgtagccgatctc-3' (nt position 1083–1102). The TaqMan probe 5'-cccaacacccagcccccgca-3' (nt position 924–943) was labeled at the 5' end with the reporter dye FAM (6-carboxy-fluorescein) and at the 3' end with the quencher TAMRA (6-carboxy-teremethyl-rhodamine) (TaKaRa). cDNA was prepared with 1 ug of total RNA from each sample using random hexamers and reverse transcription with the PrimeScriptTM RT-PCR Kit (TaKaRa). A 232 bp DNA fragment was produced by 1st strand cDNA synthesis with the above primer set, cloned into the pGEM-T Easy Vector (Promega), and verified by sequence analysis. Purified recombinant plasmid was serially diluted to 1×10^7^, 1×10^6^, 1×10^5^, 1×10^4^, 1×10^3 ^and 1×10^2 ^copies/ml. TaqMan real-time PCR was performed using the PrimeScriptTM RT-PCR Kit (TaKaRa) in 25 ul of reaction mixtures containing 12.5 ul of Premix Ex Taq, 0.5 ul of forward primer (10 uM), 0.5 ul of reverse primer (10 uM), 1 ul of TaqMan probe (3 uM), 2 ul of recombinant plasmid or cDNA and 8.5 ul of H_2_O. The following optimized thermal cycling program was used: denaturation at 95°C for 3 min, followed by 50 cycles of 95°C for 30 s and 60°C for 30 s; fluorescence data were collected at the 60°C annealing and extension step. The PCR threshold cycle (Ct), which is defined as the fractional cycle number at which the fluorescence reaches 10 times the standard deviation of the baseline, was determined by the iCycler iQ software. Standard curve equations were calculated by regression analysis of the average Ct versus the log10 of the standard copy number. Copy numbers of PADI4 mRNA in clinical samples were calculated automatically by the data analysis software.

## Results

**Figure 1:**
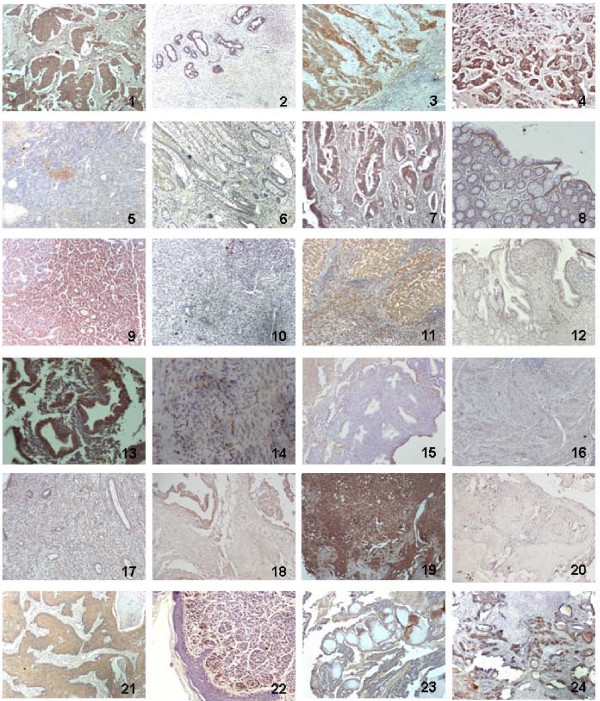
**Immunohistochemistry showing PADI4 expression in various malignant tumors, benign tumors and non-tumor inflamed tissues**. 1. breast cancer, 2. breast fibroadenoma. 3. esophageal squamous cell cancer, 4. gastric adenocarcinoma, 5. leiomyosarcoma of stomach, 6. gastritis, 7. colorectal adenocarcinoma, 8. colorectal polyps, 9. hepatocellular carcinoma, 10. hepatoblastoma, 11. liver cirrhosis, 12. cholecystitis, 13. endometrial carcinoma, 14. myoma of uterus, 15. endometrial hyperplasia of uterus, 16. cervicitis, 17. cervical polyp, 18. hydatidiform mole, 19. chondroma, 20. teratoma, 21. skin cancer, 22. intradermal nevus, 23. thyroid adenoma, 24. pleomorphic adenoma of salivary gland. The tissue structure of the section was defined by counterstaining with hematoxylin. The brown color in nevus cells was caused by pigment sediment. The color was also observable in the control sections without treatment of 1st or 2nd antibody. Original magnification was 100×.

PADI4 expression was detected by immunohistochemistry in all malignant tumor types examined including breast carcinomas, lung adenocarcinomas, hepatocellular carcinomas, esophageal squamous cancer cells, colorectal adenocarcinomas, renal cancer cells, ovarian adenocarcinomas, endometrial carcinomas, uterine adenocarcinomas, bladder carcinomas, chondromas, and other metastatic carcinomas (IRS ≥ 9). Furthermore, PADI4 was also expressed in over 40% of cells in malignant lymphomas (IRS ≥ 8). In contrast, no significant levels of PADI4 were detected in various benign tumors including leiomyomas of the stomach, uterine myomas, endometrial hyperplasias, cervical polyps, teratomas, hydatidiform moles, trophoblastic cell hyperplasias, hyroid adenomas, hemangiomas, lymph hyperplasias, schwannomas, neurofibromas, lipomas, and cavernous hemangiomas of the liver (IRS: 0–1). In breast fibroadenoma, thyroid adenoma, and pleomorphic adenoma samples, PADI4 was observed in the endothelial cells of capillaries and gland structures (IRS: 1–6), both of which mainly consist of benign tissues. PADI4 expression was not detected in most non-tumor inflamed tissues including cholecystitis, cervicitis and synovitis of osteoarthritis (IRS ≤ 1), although half of gastric ulcer and acute appendicitis samples displayed smeared PADI4 immunosignals (IRS: 3–6). In addition, more than 60% of the liver cirrhosis samples showed PADI4 expression (IRS: 3–8). PADI4 was also expressed in several mesenchymal cells in both tumor and non-tumor inflamed tissues. Immunohistochemical results are shown and listed in Figure 1 and Table 1, respectively.

**Figure 2:**
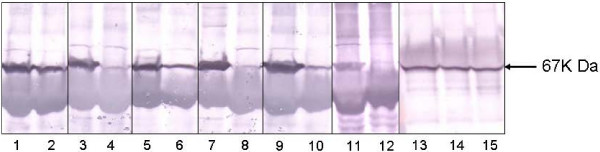
**Western blot analysis of PADI4 expression in tumor cell lines, tumors and corresponding healthy tissues**. Extracts were fractioned by electrophoresis and probed with anti-PADI4 antibody. PADI4 immunosignals (67 kDa) were detected in malignant tissues, tumor cell lines and some healthy tissues. Densities of the signals were significantly stronger in tumor tissues than in the corresponding healthy controls. Lanes 1–2, gastric adenocarcinomas and corresponding healthy tissues; lanes 3–4 lung adenocarcinomas and corresponding healthy tissues; lanes 5–6, hepatocellular carcinomas and corresponding healthy tissues; lanes 7–8, esophageal squamous cell cancers and corresponding healthy tissues; lanes 9–10, breast cancers and corresponding healthy tissues; lanes 11–12, breast fibroadenomas and corresponding healthy tissues; and lanes 13–15, A549, SKOV3 and U937 cells, respectively.

By western blot analysis, a 67 kDa band was detected in IP extracts of gastric adenocarcinomas, lung adenocarcinomas, hepatocellular carcinomas, esophageal squamous cell cancers and breast cancers as well as in their corresponding healthy tissues (see Figure 2). Immunosignal densities were significantly higher in malignant tissues than in corresponding healthy tissues or in breast fibroadenomas. Since IP extracts were purified from equivalent tissue weights for each sample, the density of the immunosignals directly corresponded to PADI4 expression levels. In addition, PADI4 was detected in total protein extracts from A549, SKOV3 and U937 tumor cell lines.

**Figure 3:**
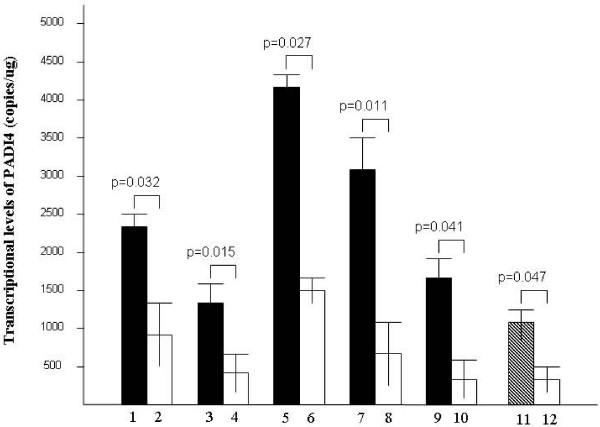
**PADI4 transcription levels in tumors and corresponding healthy tissues by TaqMan quantitative PCR**. cDNA templates were synthesized using an equivalent amount of total RNA for each sample. Lanes 1–2, PADI4 mRNA levels in gastric adenocarcinomas and corresponding healthy tissues; lanes 3–4, lung adenocarcinomas and corresponding healthy tissues; lanes 5–6, hepatocellular carcinomas and corresponding healthy tissues; lanes 7–8, esophageal squamous cancer cells and corresponding healthy tissues; lanes 9–10, breast cancer cells and corresponding healthy tissues; and lanes 11–12, breast fibroadenomas and corresponding healthy tissues.

TaqMan quantitative PCR was used to measure PADI4 transcription in tumor samples. Levels of PADI4 mRNA were determined from standard curves and amplification plots (based on the Ct and copy number). As cDNA templates were synthesized from an equivalent amount of total RNA for each sample, the copy number/ug total RNA directly corresponds to PADI4 expression levels in the tissue samples. PADI4 transcripts were detected in gastric adenocarcinomas, lung adenocarcinomas, hepatocellular carcinomas, esophageal squamous cell cancers, breast cancers and breast fibroadenomas as well as their corresponding healthy tissues. PADI4 mRNA levels were significantly elevated in malignant tissues compared to those in corresponding healthy tissues. The PADI4 mRNA level in breast fibroadenomas was lower than that in breast cancers, but higher than that in corresponding healthy tissues. TaqMan quantitative PCR results are shown in Figure 3.

**Figure 4:**
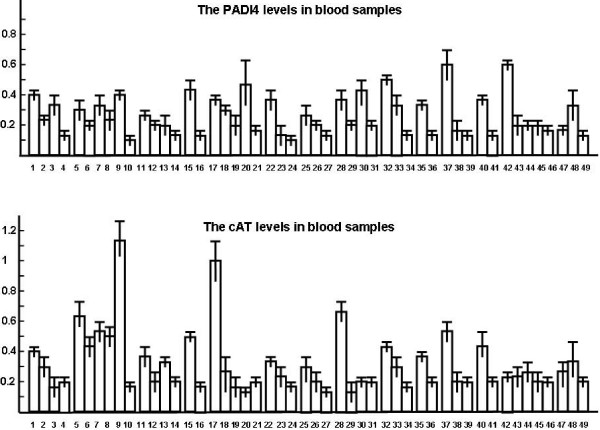
**Levels of PADI4 and cAT in plasma of patients with various tumors**. Plasma samples are listed as the followings: lane 1–4 breast carcinoma (n = 112), after receiving surgery (n = 86), breast fibroadenomas controls (n = 30), and health controls (n = 42); 5–10 hepatocellular carcinomas (n = 77), after receiving surgery (n = 24), hepatitis A controls (n = 28), hepatitis B controls (n = 14), liver cirrhosis controls (n = 27), and healthy controls (n = 42); 11–14 lung cancer (n = 127), after receiving surgery (n = 24), pulmonitis controls (n = 48), and healthy controls (n = 31); 15, 16 esophageal carcinomas (n = 64) and healthy controls (n = 44); 17–21 gastric cancer (n = 94), after receiving surgery (n = 43), chronic gastritis controls (n = 31), acute appendicitis controls (n = 22), and healthy controls (n = 38); 22–24 colon cancer (n = 21), after receiving surgery (n = 15), and healthy controls (n = 17); 25–27 rectal cancer (n = 19), after receiving surgery (n = 28), and healthy controls (n = 17); 28–31 pancreatic cancer (n = 21), after receiving surgery (n = 6), acute pancreatitis controls (n = 7), and healthy controls (n = 60); 32–34 ovarian carcinomas (n = 29), after receiving surgery (n = 11), and healthy controls (n = 23); 35, 36 prostate cancer (n = 18) and healthy controls (n = 23),; 37–39 renal cell carcinomas (n = 19), chronic nephritis controls (n = 11), and healthy controls (n = 60); 40, 41 bladder carcinomas (n = 10) and healthy controls (n = 23); 42–46 cervical cancer (n = 24), endometrial carcinomas (n = 15), uterine myomas (n = 34), cervicitis (n = 15), and healthy controls (n = 34); 47–49 thyroid carcinomas (n = 7), thyroid adenomas (n = 16), and healthy controls (n = 36).

Plasma PADI4 levels of patients with malignant tumors were measured by ELISA and compared with those with benign and healthy controls. PADI4 levels were significantly increased in the blood of patients with breast carcinomas, hepatocellular carcinomas, lung cancer, esophageal carcinomas, gastric cancer, colon cancer, rectal cancer, pancreatic cancer, ovarian carcinomas, renal cell carcinomas, cervical cancer, prostate carcinomas and bladder carcinomas (p < 0.01), but not in those with endometrial carcinomas, uterine myomas and thyroid carcinomas. After tumor excision surgery, PADI4 levels in the blood decreased considerably in breast carcinomas, hepatocellular carcinomas, lung cancer, gastric cancer, colon cancer, rectal cancer and pancreatic cancer samples (p < 0.05). Meanwhile, plasma PADI4 levels in patients with chronic gastritis, chronic nephritis and cervicitis did not considerably change, or even declined, compared to samples from healthy control patients. However, patients with breast fibroadenomas, thyroid adenomas, hepatitis A and B, liver cirrhosis, pulmonitis, acute pancreatitis and acute appendicitis had higher plasma PADI4 levels than those of healthy controls, with some samples showing even higher levels than that observed for patients with malignancies. ELISA results of PADI4 are shown in Figure 4.

Plasma cAT levels of patients with malignant tumors were measured by sandwich ELISA and compared to those with benign tumors and healthy controls. Plasma cAT levels were significantly elevated in most patients with malignant tumors, including those with breast carcinomas, hepatocellular carcinomas, lung carcinomas, esophageal carcinomas, gastric cancer, colon cancer, rectal cancer, pancreatic cancer, ovarian carcinomas, bladder carcinomas, uterine myomas, thyroid carcinomas and prostate carcinomas (p < 0.01), but not in those with cervical cancer or endometrial carcinomas. After tumor excision surgery, cAT levels in the blood declined for most malignancies. Plasma cAT expression was not considerably altered in patients with breast fibroadenomas, acute pancreatitis, chronic nephritis and cervicitis, and displayed slightly diminished levels in patients with chronic gastritis and acute appendicitis when compared to those of healthy control subjects. The cAT content was higher in samples from patients with thyroid adenomas, hepatitis A and B, liver cirrhosis and pulmonitis compared to healthy controls (see Figure 4). cAT levels associated significantly with PADI4 levels in patients with hepatocellular carcinomas, lung cancer, ovarian cancer, endometrial carcinomas and thyroid adenomas (p < 0.01). In addition, cAT levels clearly associated with CEA levels in the blood of patients with breast carcinomas (p = 0.032), CA199 levels in those with gastric cancer (p = 0.025), CEA levels in those with pancreatic cancer (p = 0.05), CA125 levels in those with ovarian cancer (p = 0.03), CEA levels in those with bladder cancer (p = 0.016), CEA in those with renal cell carcinomas (p = 0.046), and PSA in those with prostate cancer (p = 0.028). PADI4 levels also significantly associated with CEA levels in patients with gastric cancer (p = 0.05) and prostate cancer (p = 0.028).

## Discussion

We previously detected intense PADI4 expression in a variety of adenocarcinomas, but expression was absent in normal, healthy tissues [3]. In the current study, we demonstrated the presence of extensive PADI4 expression in malignant tissues, but not in most benign and non-tumor tissues. Furthermore, quantitative PCR and western blot analysis revealed significantly higher PADI4 mRNA and protein levels in malignant tissues compared to surrounding healthy tissues, which were consistent with immunohistochemical results. These findings indicated that PADI4 expression was increased in malignancies at both the transcriptional and translational levels. Additionally, PADI4 expression was detected by western blot in cultured A549, SKOV3 and U937 cancer cell lines. PADI4 expression was also detected by Abcam in cultured tumor cells such as HeLa, Jurkat, A431, HEK293, and HepG2 cells http://www.abcam.com/index.html?datasheet=38772. These results further confirmed PADI4 expression in various tumor types.

Here, relatively high PADI4 expression was observed in breast fibroadenomas and thyroid adenomas by immunohistochemistry. Co-localized PADI4 and CD34 expression in tumors, bone marrow as well as other healthy tissues suggested that cells expressing PADI4 may have originated from CD34+ stem cells [3]. As many adenomas contain more CD34+ cells [9-11], we accordingly observed extensive expression of the PADI4 enzyme in these tissues. Smeared expression of PADI4 was also detected in some non-tumor inflamed tissues such as rodents, acute gastritis and acute appendicitis. As neutrophils, monocytes, and macrophages are all derived from CD34+ stem cells and infiltrate injured tissues during inflammation [12], we expected and were able to detect PADI4 expression in these inflamed tissues. However, we note that the enzyme was predominantly located in tumor cells of malignant tissues.

Compared with healthy control subjects, high PADI4 levels were detected in the blood of patients with malignant tumors, which was consistent with results obtained by immunohistochemistry. Plasma PADI4 levels in patients with malignancies were considerably diminished after tumor excision surgery, suggesting that PADI4 protein circulates in the blood from corresponding tumor tissues. High PADI4 content was observed in breast fibroadenoma and thyroid adenoma samples, consistent with immunohistochemical results of adenomas. Although increased PADI4 levels in the blood were demonstrated in patients with acute pancreatitis and appendicitis, most patients with non-tumor inflammations, such as chronic gastritis, chronic nephritis, and cervicitis, did not exhibit variations in PADI4 levels, suggesting that PADI4 may be elevated in the blood under conditions of acute but not chronic inflammation.

High levels of cAT were detected in blood of patients with malignant tumors. Compared with PADI4 expression in the malignant tumors, cAT expression appeared to be more specific to malignant tumors and significantly associated with known tumor markers. Antithrombin is a primary plasma inhibitor of thrombin. By *in vitro *incubation, we previously demonstrated that a recombinant human PADI4 protein can inactivate the thrombin-inhibitory activity of human antithrombin III through citrullination [7]. Because the cAT content in the plasma declined after tumor excision surgery and was generally associated with PADI4 levels in the blood, we propose that the high cAT levels observed in the blood was due to citrullination of PADI4 in tumor tissues. Suppressed antithrombin III activity and unregulated thrombin activity have broadly been reported in the plasma of patients with lung adenocarcinomas, renal cell carcinomas, breast cancer, malignant melanomas, colon cancer, pancreatic adenocarcinomas, and larynx squamous cell carcinomas [13-16]. Thrombin activity can promote angiogenesis by increasing transcription of the VEGF receptor as well as metastasis and hyperplasia by inducing expression of integrin β 3 [17-19]. In the present study, high cAT production was specifically and primarily detected in blood of most malignant tumor types, but not in blood of benign tumors or non-tumor diseases, suggesting that citrullination of antithrombin may be involved in promoting carcinogenesis. Therefore, it is formally possible that deregulated thrombin activity drives thrombin-related tumorigenesis, including malignant proliferation, invasion and metastasis of tumor cells, as well as abnormal angiogenesis and fibrin deposition in tumor tissues.

**Figure 5:**
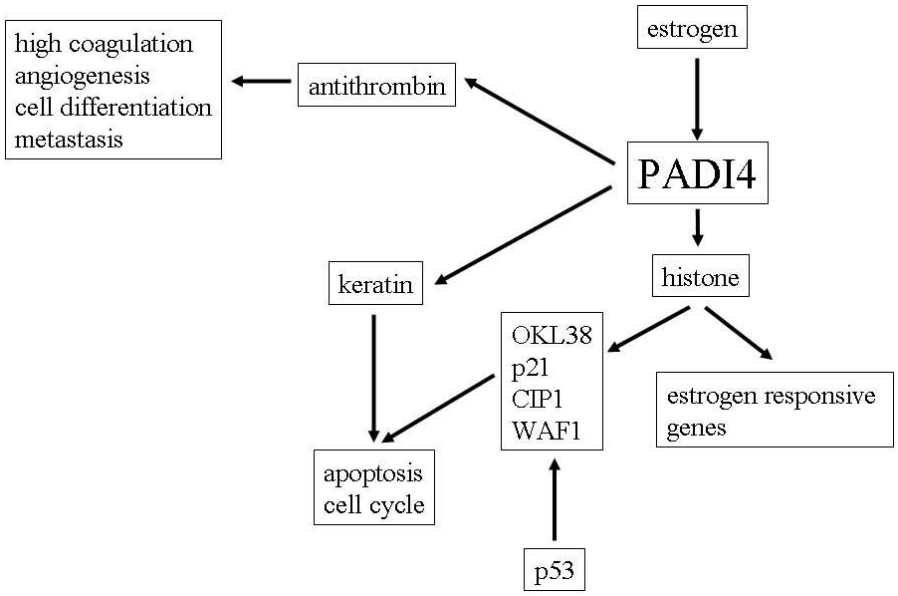
**Proposed mechanism of PADI4 for tumorigenesis**.

Many studies have demonstrated the effects of hormone metabolism on tumorigenesis [20-24]. Using cultured MCF-7 cells originated from breast cancer, Dong et al. found that estrogen enhanced PADI4 transcription in an estrogen receptor-mediated manner [25]. Recently, both Yao et al. and Li et al. reported that PADI4 repressed expressions of p53 target genes, including OKL38, p21, CIP1 and WAF1, by modifying methylated arginine sites on histones H3 and H4 [26,27]. These modifications consequently interrupt apoptosis and cell cycle progression, which are primary features of tumorigenic cells. Thus, these studies suggest that PADI4 plays a role during tumorigenesis by antagonizing regulation of p53 to tumor suppressor genes. However, we detected PADI4 primarily in the cytoplasm of tumor cells in most malignant tissues as well as citrullinated cytokeratin in certain malignant tumors [3]. As keratins (notably K5, K7, K8/K18, K19, and K20) exhibit characteristic expression patterns in human tumors, and have great importance in immunohistochemical tumor diagnosis of carcinomas [6,28,29], it is thus possible that citrullination of histones, keratin and antithrombin are all involved in the tumorigenic process. Our proposed mechanism for PADI4 in tumorigenesis is shown in Figure 5.

## Conclusion

Compared to benign and non-tumor diseases, many malignant tumor types exhibited increased PADI4 expression in tumorous cells. Patients with malignant tumors also displayed higher plasma levels of both PADI4 and cAT than patients with benign and non-tumor inflammations. Plasma levels of cAT and PADI4 in patients with these malignancies were significantly associated with levels of known tumor markers. Our results implicate the importance of PADI4 and citrullination in the promotion of tumorigenesis. However, further study is needed to understand the exact pathogenic mechanism.

## Competing interests

The authors declare that they have no competing interests.

## Authors' contributions

X.C. designed the study, performed experiments and prepared the manuscript; J.H. evaluated data; Y.Z. performed experiments; L.P., Y.Y. and Z.S. collected tissue and blood samples.

## Pre-publication history

The pre-publication history for this paper can be accessed here:

http://www.biomedcentral.com/1471-2407/9/40/prepub
